# The HLA Variant rs6903608 Is Associated with Disease Onset and Relapse of Immune-Mediated Thrombotic Thrombocytopenic Purpura in Caucasians

**DOI:** 10.3390/jcm9103379

**Published:** 2020-10-21

**Authors:** Ilaria Mancini, Elisa Giacomini, Silvia Pontiggia, Andrea Artoni, Barbara Ferrari, Emanuela Pappalardo, Roberta Gualtierotti, Silvia Maria Trisolini, Saveria Capria, Luca Facchini, Katia Codeluppi, Erminia Rinaldi, Domenico Pastore, Simona Campus, Cinzia Caria, Aldo Caddori, Daniela Nicolosi, Gaetano Giuffrida, Vanessa Agostini, Umberto Roncarati, Clara Mannarella, Alberto Fragasso, Gian Marco Podda, Simone Birocchi, Anna Maria Cerbone, Antonella Tufano, Giuseppe Menna, Michele Pizzuti, Michela Ronchi, Alessandro De Fanti, Sergio Amarri, Marzia Defina, Monica Bocchia, Silvia Cerù, Salvatore Gattillo, Frits R. Rosendaal, Flora Peyvandi

**Affiliations:** 1 Department of Pathophysiology and Transplantation, Università degli Studi di Milano, and Fondazione Luigi Villa, 20122 Milan, Italy; ilaria.mancini@guest.unimi.it (I.M.); elisa.giacomini2@gmail.com (E.G.); emanuela.pappalardo@unimi.it (E.P.); roberta.gualtierotti@unimi.it (R.G.); 2 Fondazione IRCCS Ca’ Granda Ospedale Maggiore Policlinico, Angelo Bianchi Bonomi Hemophilia and Thrombosis Center, 20122 Milan, Italy; silviapontiggia@alice.it (S.P.); andrea.artoni@policlinico.mi.it (A.A.); barbara.ferrari@policlinico.mi.it (B.F.); 3 Hematology, Department of Translational and Precision Medicine, “Sapienza” University of Rome, 00161 Rome, Italy; trisolini@bce.uniroma1.it (S.M.T.); capria@bce.uniroma.it (S.C.); 4 Hematology Unit, Azienda Unità Sanitaria Locale—IRCCS di Reggio Emilia, 42122 Reggio Emilia, Italy; facchini.luca@asmn.re.it (L.F.); katia.codeluppi@asmn.re.it (K.C.); 5 Hematology Unit, A. Perrino Hospital, 72100 Brindisi, Italy; rinaldierminia79@gmail.com (E.R.); domenico.pastore0@gmail.com (D.P.); 6 Pediatric Unit, Ospedale Microcitemico, 09121 Cagliari, Italy; simona.campus1978@gmail.com; 7 Internal Medicine Unit, S.S. Trinità Hospital, 09121 Cagliari, Italy; cinziacariacc74@gmail.com (C.C.); Aldori@libero.it (A.C.); 8 Hematology Division, Department of Clinical and Molecular Biomedicine, University of Catania, 95123 Catania, Italy; danielanicolosi03@gmail.com (D.N.); gaegiuffrida@gmail.com (G.G.); 9 U.O. Medicina Trasfusionale, IRCCS—Ospedale Policlinico San Martino, 16132 Genova, Italy; Vanessa.Agostini@hsanmartino.it; 10 U.O. Immunoematologia e Medicina Trasfusionale/Officina Trasfusionale, Cesena e Forlì, 47521 Cesena, Italy; umberto.roncarati@auslromagna.it; 11 Hematology Unit, Madonna delle Grazie Hospital, 75100 Matera, Italy; mannarellaclara@asmbasilicata.it (C.M.); albertofragasso50@gmail.com (A.F.); 12 U.O. Medicina III, ASST Santi Paolo e Carlo, Dipartimento di Scienze della Salute, Università degli Studi di Milano, 20142 Milan, Italy; gmpodda@gmail.com (G.M.P.); simonebirocchi@gmail.com (S.B.); 13 Department of Clinical Medicine and Surgery, AOU Federico II, 80131 Naples, Italy; ancerbon@unina.it (A.M.C.); atufano@unina.it (A.T.); 14 Department of Oncology, AORN Santobono-Pausilipon, 80122 Naples, Italy; giumenna56@libero.it; 15 Hematology Unit, AO San Carlo, 85100 Potenza, Italy; pizzuti.m@tiscali.it; 16 Internal Medicine Unit, Department of Medicine, Lugo Hospital, Lugo, 48022 Ravenna, Italy; michela.ronchi@auslromagna.it; 17 Departmental Simple Unit of Pediatric Rheumatology, AUSL-IRCSS Reggio Emilia, 42123 Reggio Emilia, Italy; Alessandro.DeFanti@asmn.re.it; 18 Paediatrics Unit, AUSL-IRCSS Reggio Emilia, 42123 Reggio Emilia, Italy; Sergio.Amarri@ausl.re.it; 19 Department of Medical, Surgery and Neuroscience, Hematology Unit, Azienda Ospedaliera Universitaria Senese, Università degli Studi di Siena, 53100 Siena, Italy; marziadefina@libero.it (M.D.); bocchia@unisi.it (M.B.); 20 Hematology Unit, Santa Chiara Hospital, 38122 Trento, Italy; Silvia.Ceru@apss.tn.it; 21 Immuno-Hematology and Transfusion Medicine Unit, San Raffaele Hospital, 20132 Milan, Italy; gattillo.salvatore@hsr.it; 22 Department of Clinical Epidemiology, Leiden University Medical Center, 2333 ZA Leiden, The Netherlands; F.R.Rosendaal@lumc.nl

**Keywords:** ADAMTS13, autoimmune disease, genotyping, HLA, relapse, risk factor, thrombotic thrombocytopenic purpura

## Abstract

Immune-mediated thrombotic thrombocytopenic purpura (iTTP) is a rare, life-threatening thrombotic microangiopathy caused by severe ADAMTS13 (a disintegrin and metalloproteinase with thrombospondin motifs 13) deficiency, recurring in 30–50% of patients. The common human leukocyte antigen (HLA) variant rs6903608 was found to be associated with prevalent iTTP, but whether this variant is associated with disease relapse is unknown. To estimate the impact of rs6903608 on iTTP onset and relapse, we performed a case-control and cohort study in 161 Italian patients with a first iTTP episode between 2002 and 2018, and in 456 Italian controls. Variation in rs6903608 was strongly associated with iTTP onset (homozygotes odds ratio (OR) 4.68 (95% confidence interval (CI) 2.67 to 8.23); heterozygotes OR 1.64 (95%CI 0.95 to 2.83)), which occurred over three years earlier for each extra risk allele (β −3.34, 95%CI −6.69 to 0.02). Of 153 survivors (median follow-up 4.9 years (95%CI 3.7 to 6.1)), 44 (29%) relapsed. The risk allele homozygotes had a 46% (95%CI 36 to 57%) absolute risk of relapse by year 6, which was significantly higher than both heterozygotes (22% (95%CI 16 to 29%)) and reference allele homozygotes (30% (95%CI 23 to 39%)). In conclusion, HLA variant rs6903608 is a risk factor for both iTTP onset and relapse. This newly identified biomarker may help with recognizing patients at high risk of relapse, who would benefit from close monitoring or intensified immunosuppressive therapy.

## 1. Introduction

Thrombotic thrombocytopenic purpura (TTP) is a rare and life-threatening thrombotic microangiopathy which manifests as an acute episode of thrombocytopenia, microangiopathic hemolytic anemia and disseminated microvascular thrombosis with variable and multiple organ involvement [1]. TTP is caused by a severe deficiency of the von Willebrand factor cleaving protease ADAMTS13 (a disintegrin metalloproteinase with thrombospondin type 1 motif 13) and exists in two forms: congenital TTP, an autosomal recessive disease due to mutations in the ADAMTS13 gene on chromosome 9, and acquired immune-mediated TTP (iTTP), an autoimmune disease caused by immunological tolerance loss in ADAMTS13. With an estimated annual incidence of 2–6 cases per million people [2,3], iTTP affects mostly women in the forth to fifth decade of life [4,5], and may arise idiopathically or secondary to other conditions, such as concomitant autoimmune diseases and pregnancy [1]. Despite standard treatment with plasma exchange and immunosuppressors, mortality (about 10%) [6,7], exacerbation of acute TTP (up to 50% of cases) [6], major thrombotic complications and refractoriness to standard therapy [8] are challenges of acute iTTP management. Notably, up to 50% of patients develop one or more acute TTP relapses [6] which, although often milder than the first events [9], expose patients to the same aforementioned risks. Severe ADAMTS13 deficiency and the presence of anti-ADAMTS13 autoantibodies during disease remission are known risk factors for iTTP relapse [10,11], which, however, remains largely unpredictable.

Similarly to other autoimmune diseases, a genetic contribution to iTTP has been described, particularly of the human leukocyte antigen (HLA) class II locus. The HLA allele DRB1*11 has been consistently reported as a risk factor in European Caucasian populations [12,13,14,15,16], and CD4+ T-cells reactive to specific ADAMTS13 CUB2 domain-derived peptides were found in HLA-DRB1*11-positive iTTP patients [17,18]. Furthermore, in a recent case-control genetic association study, our group identified the common, intergenic HLA variant rs6903608 to be independently associated with prevalent iTTP in Italians (minor allele frequency in controls 0.47, pooled odds ratio (OR) and 95% confidence interval (CI) of discovery and replication phases 2.48, 2.03 to 3.02, P 3.95 × 10^−19^) [16]. Whether or not these genetic markers also predict iTTP relapse is unknown.

With this background, the aim of this study was to precisely estimate the impact of the HLA variant rs6903608 on the onset (i.e., the first event) of iTTP, and to assess whether this novel marker also predicts relapse, the prevention of which represents one of the most urgent needs in iTTP care.

## 2. Materials and Methods

### 2.1. Study Design, Subjects and Definitions

As the purpose of our previous study was to identify novel genetic risk factors for iTTP across immune-related areas of the genome, we enrolled all eligible patients available at that time, regardless of the number of acute TTP events they had experienced [16]. Therefore, the risk estimate that we found for the HLA variant rs6903608 (allelic OR 2.48) was most probably a combination of at least three possible scenarios of association: (1) rs6903608 increases the risk of a first episode of iTTP but not that of disease relapse; (2) rs6903608 has no effect on a first episode of iTTP but it increases the risk of iTTP relapse; (3) rs6903608 increases the risk of both a first episode of iTTP and relapse (Supplemental Figure S1). In order to dissect the association, a combination of case-control and cohort study design was applied to all iTTP patients referred to our center for ADAMTS13 testing for a first episode of iTTP between the 1st of January 2002 and the 15th of March 2018. First, we evaluated the association of the HLA variant rs6903608 with the occurrence of a first iTTP episode (case-control design), then with a first iTTP relapse (cohort design). In the case-control phase of our study, sex was analyzed as an effect modifier. Secondary analyses included the occurrence of a first episode of iTTP in association with (i) other autoimmune diseases (diagnosed before or during the iTTP event), or (ii) infectious triggers reported within three months before the acute event. Furthermore, we analyzed the impact of the rs6903608 risk allele on the age of iTTP onset.

Patients were selected from the TTP register kept at our institution (Milan TTP registry, www.ttpdatabase.org). The diagnosis of iTTP was based on microangiopatic hemolytic anemia with thrombocytopenia, in the absence of alternative causes [19], such as active neoplasm, bone marrow transplantation and active HIV infection. Diagnosis of iTTP had to be confirmed by the evidence of severe ADAMTS13 deficiency (ADAMTS13 activity levels below 10% of normal) and anti-ADAMTS13 antibodies in at least one sample collected during the acute or remission phase, throughout the time of patient observation. The exclusion criteria included non-Italian birth, non-Caucasian ethnicity and unavailability of a DNA sample. Clinical and laboratory data, including those regarding concomitant autoimmune diseases, infectious triggers and treatment, were collected through a standardized questionnaire. Each investigator provided the information based on the disease history given by the patient and the patient clinical examination during the hospitalization/diagnostic process and follow-up, according to routine clinical practice. The latter includes full autoimmune laboratory testing panels (such as complement C3 and C4, antinuclear, anti-ENA and anti-DNA antibodies, autoimmune vasculitis and thyroiditis panels, antiphospholipids antibodies) and familiar anamnesis for the presence or absence of autoimmune conditions. With regards to infections, as per clinical practice, the screening included testing of reactive C protein, serology for major hepatic viruses, HIV antibodies, chest X-ray and abdominal ultrasound.

For the purpose of the case-control study, 456 healthy Italian individuals genotyped in the replication phase of our previous study were included as controls [16]. These were healthy volunteers recruited between 2006 and 2014 among friends and non-consanguineous relatives of patients tested for thrombophilia at the Hemophilia and Thrombosis Center of Milan. For the purpose of the cohort study, patients with a fatal first acute iTTP episode or with missing information soon after the first iTTP episode were excluded. Follow-up started from the date of remission of the first iTTP episode to the date of relapse, death, cancer diagnosis, last contact or the 15th of March 2018, whichever came first. Remission was defined as the absence of signs and symptoms of acute TTP for at least 30 days after sustained platelet normalization (≥150 × 10^9^/L) for 48 consecutive hours, whereas relapse was defined as the re-occurrence of an acute TTP episode after having reached remission of the previous episode [1,19]. Relapse was confirmed by the detection of severe ADAMTS13 deficiency.

Written informed consent was obtained from all subjects with the approval of the Ethics Committee of our institution (approval numbers 1163-09/06/2005, 123-22/01/2014), in accordance with the Declaration of Helsinki.

### 2.2. rs6903608 Genotyping

The genotyping of variant rs6903608 (C/T) was performed with SNP TaqMan assays (Thermo Fisher Scientific, Carlsbad, CA, USA) and the high-performance StepOnePlus Real-Time PCR System (Thermo Fisher Scientific), according to the manufacturer’s instructions. In the present study, the rs6903608 C and T alleles are referred to as the risk and reference allele, based on the frequencies observed in cases and controls in our previous study [16].

### 2.3. Statistical Analysis

Categorical variables were expressed as counts and percentages, and continuous variables as medians and interquartile ranges (IQR). Subjects were categorized as homozygous for the risk allele (CC), or heterozygous (CT) or homozygous for the reference allele (TT). The relationship between exposure and study outcomes was analyzed assuming either an additive (CC versus TT, CT versus TT), a dominant (CC plus CT versus TT), or a recessive (CC versus CT plus TT) model of inheritance. Pertaining to the case-control study, logistic regression analysis was used to estimate the risk of a first episode of iTTP for the various genotypes. Effect modification by sex was assessed by stratification. Differences between means with 95% CI were estimated using an independent samples *t*-test to compare the age at disease onset in iTTP patients with different rs6903608 genotypes. The relationship between genotype and age of iTTP onset was also studied by linear regression analysis.

With regard to the cohort study, the cumulative incidence of relapse by genotype was estimated by Kaplan–Meier survival techniques, with 95% CI calculated by the Hosmer–Lemeshow–May method. Median follow-up time was estimated by the reverse Kaplan–Meier method [20]. Cox regression analysis was performed to estimate the relative risk of a relapse of iTTP in patients with the risk genotype compared with the reference genotype, before and after adjusting for age at iTTP onset. In the main analysis, time to event was calculated from the date of remission of the first acute episode, as stated above. For patients in whom the exact day of remission was unknown, we assumed an episode duration of 30 days based on the distribution of time to remission in subjects with available information (median 18 days, IQR 8 to 27). A secondary analysis calculating time to event from the day of acute onset was also performed to ensure that our results were not affected by missing data, and this led to the same results. To account for the influence of rituximab on the risk of relapse, we performed the following analyses: (1) Cox regression adjusting also for rituximab use; (2) Cox regression after restriction to patients who were never treated with rituximab, assuming a recessive model of inheritance due to power issues. Finally, a sensitivity analysis for loss to follow up was performed assuming the following two extreme scenarios: (i) patients lost to follow-up had a recurrence on the last observation day, and (ii) patients lost to follow-up were censored alive at the end of the study in 2018.

Risk estimates were expressed as OR, beta, or hazard ratios (HR) with 95% CI as results of logistic, linear and Cox regression analyses, respectively.

Power calculations were performed assuming a risk of 2, an alpha error of 0.05 and a beta error of 0.20 (a priori sample size calculation), or the actual sample size (post-hoc power calculation).

Statistical analyses were performed by SPSS, release 25.0 (IBM Corp., Armonk, NY, USA), and GraphPad Prism, version 7.03 (GraphPad Software, La Jolla, CA, USA).

## 3. Results

**Figure 1:**
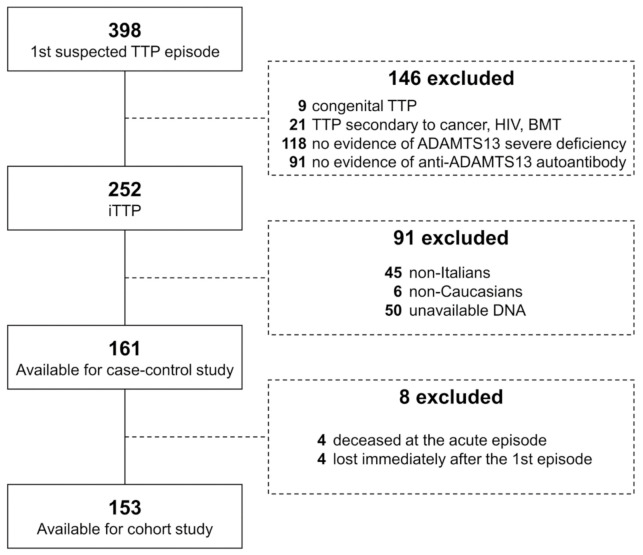
Study flow-chart. A patient could have been excluded as a result of more than one criterion. BMT, bone-marrow transplant; HIV, human immunodeficiency virus; iTTP, immune-mediated thrombotic thrombocytopenic purpura.

Between January 2002 and March 2018, 252 patients were referred to the Angelo Bianchi Bonomi Hemophilia and Thrombosis Center for ADAMTS13 testing for a first episode of iTTP. Among them, 161 were eligible for this study, as depicted in Figure 1. 

The majority of patients were women (77%), with a median age at the first iTTP episode of 45 years (IQR 33 to 54, range 11 to 79 years) (Table 1).

In total, 74 (46%) iTTP patients were homozygous for the risk allele (CC), and 67 (42%) were heterozygous (CT) and 20 (12%) homozygous for the reference allele (TT) (Table 2). Of the 161 eligible patients, 52 were newly genotyped after our previous study. The risk allele frequency in new cases was 0.663, similar to previous estimates (0.696 and 0.665 in discovery and replication phases, respectively) [16].

Table 2 reports the results of the association analyses between the rs6903608 genotype and case-control status, assuming a genotypic, dominant or recessive model of inheritance. Variant rs6903608 was strongly associated with the occurrence of a first episode of iTTP (OR of homozygous subjects for the risk allele (CC) versus homozygous subjects for the reference allele (TT): 4.68 (95% CI 2.67 to 8.23); OR of heterozygous subjects (CT) versus homozygous subjects for the reference allele (TT): 1.64 (95% CI 0.95 to 2.83)). The results of association obtained by assuming different inheritance models supported the additive effect of the risk allele. Stratified analysis by sex revealed a risk increase for iTTP by each extra allele for both men and women (Table 3).

Given the immune-related function of the genomic area of the analyzed variant, the association with a first iTTP episode in the absence or presence of concomitant autoimmune diseases or infectious triggers was also evaluated. A concomitant autoimmune disorder was diagnosed before or during the first acute TTP event in 19 (12%) patients, whereas in 37 (24%) an infection preceding the event was reported (Supplemental Table S1). The increased risk conferred by the HLA variant rs6903608 was confirmed among patients with no concomitant autoimmune disorder (homozygous risk genotype: OR 6.06 (95% CI 3.21 to 11.45); heterozygous genotype: OR 2.13 (95% CI 1.15 to 3.97)), while the risk virtually disappeared in cases diagnosed with another autoimmune disease before or during the first acute iTTP event (homozygous risk genotype: OR 1.48 (95% CI 0.48 to 4.54); heterozygous genotype: OR 0.49 (95% CI 0.16 to 1.55)) (Table 4). Conversely, neither the presence nor the absence of infectious triggers affected the risk estimates of developing iTTP according to the rs6903608 genotype (Table 4).

As expected for a genotype that increases the risk, patients with the risk genotype developed iTTP earlier than those with the reference genotype (mean difference 7 years (95% CI 0 to 14 years)) (Table 5). With linear regression we found an over three-years-earlier onset as a result of an extra risk allele (β −3.34, 95% CI −6.69 to 0.02).

In total, 153 survivors with available follow-up were eligible for the cohort study (Figure 1). The follow-up features are in Table 6. The median follow-up duration was 4.9 years (95% CI 3.7 to 6.1 years). In total, 29% of the patients (17% of reference allele homozygotes, 20% of heterozygotes and 41% of risk allele homozygotes) experienced a relapse, occurring within four years of the first acute event in 91% of the cases. 

**Figure 2:**
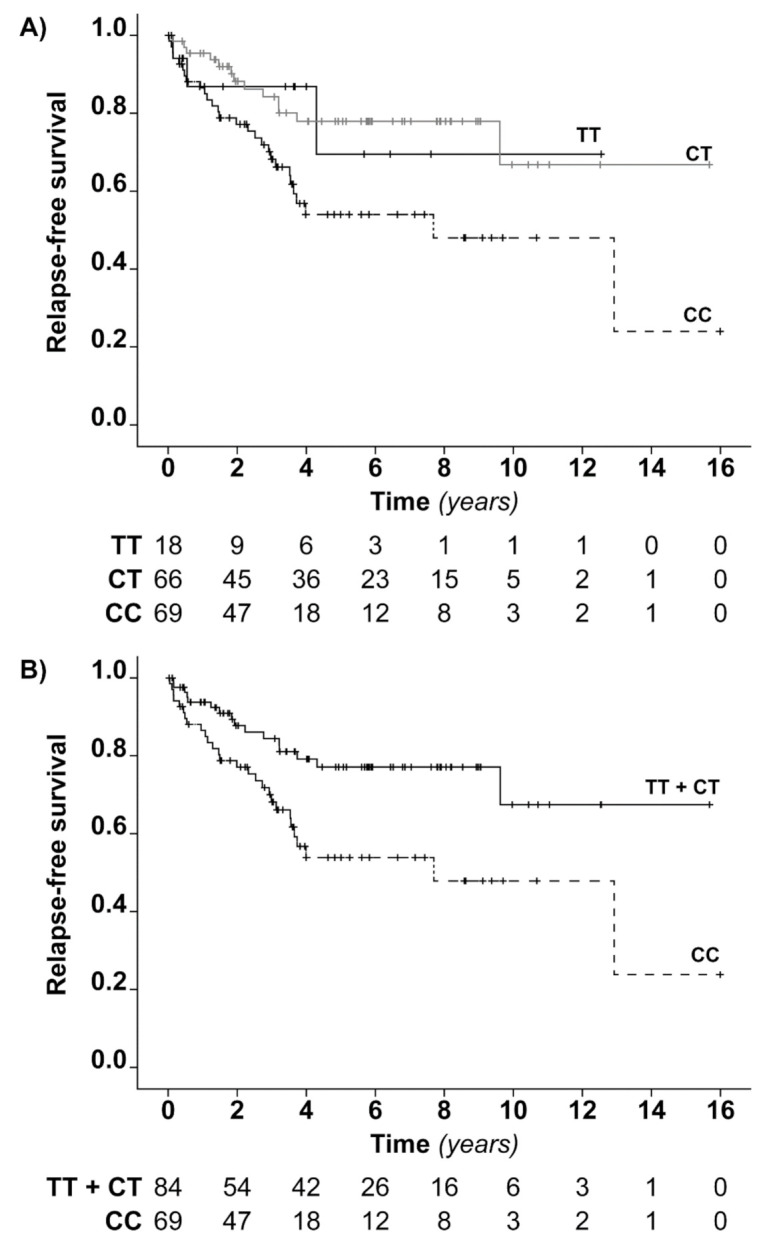
Relapse-free survival analysis in iTTP patients according to the rs6903608 genotype. (**A**) The Kaplan–Meier curves show the cumulative probability of being relapse-free in iTTP patients homozygous for the risk allele (CC), heterozygous (CT) and homozygous for the reference allele (TT). (**B**) Kaplan–Meier curves show the cumulative probability of being relapse-free in iTTP patients homozygous for the risk allele (CC) and carriers of reference allele (TT + CT). Tick-marks indicate censored subjects. Below the figures, the numbers of subjects at risk of TTP relapse at 0, 2, 4, 6, 8, 10, 12, 14 and 16 years from the remission of the first acute episode are reported.

Kaplan–Meier curves depicting relapse-free survival in iTTP patients according to the rs6903608 genotype are shown in Figure 2. The cumulative incidence of relapse at 6 years was 30% (95% CI 23% to 39%) for homozygotes for the reference allele, 22% (95% CI 16 to 29%) for heterozygotes, and 46% (95% CI 36 to 57%) for patients homozygous for the risk allele (Table 7 and Figure 2A). The latter group of patients had an over twofold increased rate of relapse compared with both heterozygotes (unadjusted HR 2.42 (95% CI 1.25 to 4.69), age-adjusted HR 2.43 (95% CI 1.25 to 4.71)) and reference allele homozygotes (HR 2.07 (95% CI 0.63 to 6.82), age-adjusted HR 2.12 (95% CI 0.64 to 7.07)). No difference was observed between heterozygotes and patients homozygous for the reference allele, most probably due to low power. Indeed, a sample size calculation indicates the need for 68 subjects per arm to detect a relative rate of 2—many more than the 18 patients with the rs6903608 TT genotype that were actually enrolled. Overall, the small number of cases is reflected in the wide confidence intervals of some of the estimates resulting from both the case-control and cohort study analyses. 

Due to the low number of patients and events in the reference genotype group, we also analyzed the relapse free survival of iTTP survivors according to a recessive model of inheritance (Figure 2B). The Kaplan–Meier curves showed a relapse rate that was higher in patients homozygous for the risk allele than in carriers of the reference allele, with an HR for relapse of 2.37 (95% CI 1.28 to 4.40; age-adjusted HR 2.39 (95% CI 1.29 to 4.44)), as estimated by Cox regression.

We had available information on rituximab treatment in 148 out of the 153 patients of the cohort study—19 patients (13%) were treated with rituximab during the course of the acute TTP event, 9 (6%) were treated during disease remission and 10 (7%) were treated with rituximab during both the acute and the remission phase of the disease. To account for the influence of rituximab on the risk of relapse, we performed Cox regression, adjusting also for rituximab use, and Cox regression after restriction to the 110 patients who were never treated with rituximab, assuming a recessive model of inheritance due to power issues (50 patients homozygous for the risk allele versus 60 carriers of the reference allele). The HRs were similar to those obtained in our main analysis (Supplemental Table S2), demonstrating that treatment with rituximab does not affect our results.

Finally, we performed a sensitivity analysis to account for the 11 (7%) iTTP patients lost to follow-up. In either scenario, the increased risk of relapse conferred by the rs6903608 risk genotype was confirmed, as follows: (1) all those lost to follow-up had a relapse on the same day in which they were lost, with an HR of rs6903608 CC patients versus CT and TT patients of 2.25 (95% CI 1.30 to 3.90); (2) all lost to follow-up were censored alive at the end of the study, with an HR of rs6903608 CC patients versus CT and TT patients of 2.34 (95% CI 1.26 to 4.33). 

## 4. Discussion

In a large population of 161 Italian Caucasian incident cases of iTTP, we found that the high-risk rs6903608 genotype was associated with both the first and the second episode of acute iTTP, conferring a 4.7-fold (95% CI 2.7 to 8.2) and a 2.1-fold (95% CI 0.6 to 7.1) higher risk than the reference genotype, respectively. Furthermore, the rs6903608 variant predisposed the patient to an earlier disease onset of about 3 years per risk allele (95% CI−7 to 0 years).

Given the severity and life-threatening nature of iTTP, an estimated increase in risk of developing the disease of almost five-fold could be considered of clinical importance. However, the low incidence of iTTP (two cases per million per year) [2] and the common frequency of the high-risk genotype do not make rs6903608 a potential candidate for genetic screening to identify high-risk subjects regarding first events. However, given the substantial difference in absolute risks of relapse, the variant may prove useful once iTTP has been diagnosed, in combination with other markers of recurrence already available in the clinic (i.e., severe ADAMTS13 deficiency and positive anti-ADAMTS13 autoantibodies during remission) [10,11], in order to identify patients at high risk of iTTP relapse, who may benefit from a close monitoring or an intensified therapy.

Female sex and genetic variation at the HLA locus are known risk factors for autoimmunity. It is unknown whether there are sex-specific associations between iTTP and HLA alleles, which have, for instance, been reported in multiple sclerosis [21,22]. A differential impact of the HLA-DRB1-DQB1 haplotypes on disease risk between male and female TTP patients was recently described by Sinkovits and colleagues, although their limited number of patients did not allow them to draw any conclusions [15]. In the present study, we found an increased risk of a first episode of iTTP in both men and women carriers of the rs6903608 risk variant, which was about 10-fold in homozygous individuals (95% CIs 2.7 to 61.1 and 2.2 to 43.6 in men and women, respectively). The small number of patients with both risk factors and large confidence intervals does not allow us to infer more. Further analyses on larger patient populations are needed to investigate a link between genetic variation in the HLA locus and the well-recognized increased predisposition of women to developing iTTP.

When we restricted the case-control analysis to iTTP patients without concomitant autoimmune diseases, the association was similar on a relative scale (OR 6.1 versus 4.7 overall). Conversely, the effect was much smaller when we restricted the analysis to cases with a concomitant autoimmune disorder (OR 1.5 versus 4.7 overall). These results might be merely due to the known increased risk of iTTP conferred by concomitant autoimmune diseases, or they might indicate an iTTP-specific mechanism of action, enacted by the variant rs6903608 and not shared by other autoimmune disorders that commonly co-manifest in these patients. However, the small sample size of the latter group of patients failed to ensure sufficient power to draw conclusions on the interaction of other autoimmune disorders with the rs6903608 genotype. Differently from other autoimmune diseases, the presence or absence of infections before the first acute iTTP event did not influence the effect of rs6903608. A potential explanation for this is that rs6903608 is located in the HLA class II (involved in self antigens presentation) and not in the HLA class I locus (involved in foreign antigens presentation).

The functional effect of rs6903608 on iTTP development is unknown. In silico analysis suggested that rs6903608 and/or its haplotype might increase the expression of nearby HLA genes, potentially favoring autoimmunization via the enhanced presentation of specific peptide(s) derived from the autoantigen ADAMTS13 to CD4+ T-cells [16]. Immunochip genotyping and HLA-imputed data from our previous study also indicated the high linkage disequilibrium between rs6903608 and the high-risk allele HLA-DRB1*11 in Italians [16]. Whether this variant has a regulatory function itself, or merely tags HLA-DRB1*11 or a causal variant elsewhere in the HLA locus, it does not invalidate the translational value of our findings. Furthermore, SNP genotyping would be much easier and cheaper than HLA typing.

The main limitation of this study is represented by referral. Our center is a highly-specialized tertiary-care center and our population might be either (i) enriched with those with a more severe disease, harder to treat in smaller unexperienced hospitals, and (ii) lacking in those who died before reaching our attention (immortality bias). However, as the reason for referral cannot be linked to genetic factors, especially not to an HLA variant which was unknown until recently, we can confidently exclude the consideration that referral may have influenced our results. Secondly, we enrolled only patients referred to us after having had a first episode of TTP, reducing the bias introduced by survivorship. Another limitation for the cohort study was the small sample size, especially regarding patients homozygous for the reference allele, that reflects both the rarity of iTTP and the low frequency of the reference allele among patients (T allele frequency 0.33). We calculated the post hoc power to detect a relative risk of relapse of 2, based on the available sample size (69 CC and 18 TT patients). The power to detect significant signals was less than 40%, not allowing us to fully dissect the risk associated with each genotype. However, it is worth pointing out that iTTP is a very rare disorder, making it challenging to achieve larger sample sizes. A further limitation of our study resides in the variable frequency of this variant across different populations, with a minor allele frequency of rs6903608 in the gnomAD database (v2.1.1, http://gnomad.broadinstitute.org, last access 02/09/2020) ranging from 0.22 in the European Finnish population to 0.44 in the East Asian and southern European populations. Although similar to the southern Europeans estimate, the minor allele frequency we found in Italian controls (0.47) [16] is higher than the global frequency across all populations, indicating that further studies are needed to evaluate the generalizability of our findings to other populations. Furthermore, the impact of variant rs6903608, as well as that of HLA allele DRB1*11, on susceptibility to iTTP appears to be limited to Caucasian European populations, as recently reported by Sakai et al., who did not find an association in Japanese patients [23].

In summary, we have found that the HLA variant rs6903608 is associated with both the occurrence of a first episode and a relapse of iTTP in Italian Caucasian patients, with high absolute risks of relapse. Although a larger sample size is needed to precisely estimate the effect of each additional rs6903608 risk allele on iTTP relapse, this genetic marker may help to identify patients at higher risk who would benefit from a closer follow-up or a more intensive immunosuppressive therapy.

## Figures and Tables

**Table 1:** Demographic and clinical features of cases included in the study.

Variable	Value (*n* = 161)
Female sex, *n* (%)	124 (77)
Age at first iTTP episode, median (IQR)	45 (33 to 54)
Clinical characteristics	
Concomitant autoimmune disease, *n* (%) *	19 (12)
Infectious trigger, *n* (%)	37 (24)
Platelet count, ×10^9^/L, median (IQR) †	13 (8 to 20)
Hemoglobin, g/dL, median (IQR) †	8.0 (6.9 to 9.6)
LDH, IU/L, median (IQR) †	1418 (748 to 2340)

IQR, interquartile range; iTTP, immune-mediated thrombotic thrombocytopenic purpura; LDH, lactate dehydrogenase. * Diagnosed before or during the acute iTTP episode. † At presentation of the first acute iTTP episode, before any plasma treatment. Available for 154 (platelet count, hemoglobin) and 145 (LDH) patients.

**Table 2:** Risk of a first iTTP episode by rs6903608 genotype.

rs6903608 Genotype	Cases (*n* = 161)	Controls (*n* = 456)	OR (95% CI)
Genotypic model			
TT, *n* (%)	20 (12)	119 (26)	Reference
CT, *n* (%)	67 (42)	243 (53)	1.64 (0.95 to 2.83)
CC, *n* (%)	74 (46)	94 (21)	4.68 (2.67 to 8.23)
Dominant model			
TT, *n* (%)	20 (12)	119 (26)	Reference
CT + CC, *n* (%)	141 (88)	337 (74)	2.49 (1.49 to 4.16)
Recessive model			
TT + CT, *n* (%)	87 (54)	362 (79)	Reference
CC, *n* (%)	74 (46)	94 (21)	3.28 (2.23 to 4.81)

rs6903608 genotype: TT, homozygous reference; CT, heterozygous; CC homozygous risk; OR, odds ratio; CI, confidence interval.

**Table 3:** Stratified analysis by sex.

Sex	rs6903608 Genotype	Cases (*n* = 161)	Controls (*n* = 456)	OR (95%CI)
Male, *n* (%)	TT	2 (1)	27 (6)	Reference
	CT	14 (9)	71 (16)	2.66 (0.57 to 12.50)
	CC	21 (13)	22 (5)	12.89 (2.72 to 61.07)
Female, *n* (%)	TT	18 (11)	92 (20)	2.64 (0.58 to 12.11)
	CT	53 (33)	172 (38)	4.16 (0.96 to 18.07)
	CC	53 (33)	72 (16)	9.94 (2.26 to 43.63)

rs6903608 genotype: TT, homozygous reference; CT, heterozygous; CC homozygous risk. OR, odds ratio; CI, confidence interval.

**Table 4:** Subgroup analyses by concomitant autoimmune disorder and infectious trigger preceding the acute event.

	Concomitant Autoimmune Disorder				Infectious Trigger *				
	Absent (*n* = 142)		Present (*n* = 19)		Absent (*n* = 119)		Present (*n* = 37)		Controls (*n* = 456)
rs6903608	*n* (%)	OR (95%CI)	*n* (%)	OR (95%CI)	*n* (%)	OR (95% CI)	*n* (%)	OR (95%CI)	*n* (%)
TT	14	Ref.	6	Ref.	16	Ref.	4	Ref.	119 (26)
CT	61	2.13 (1.15 to 3.97)	6	0.49 (0.16 to 1.55)	50	1.53 (0.84 to 2.80)	14	1.71 (0.55 to 5.32)	243 (53)
CC	67	6.06 (3.21 to 11.45)	7	1.48 (0.48 to 4.54)	53	4.19 (2.25 to 7.80)	19	6.01 (1.98 to 18.28)	94 (21)

rs6903608 genotype: TT, homozygous reference; CT, heterozygous; CC homozygous risk. CI, confidence interval; OR, odds ratio; Ref., reference. * Information on the trigger of the first iTTP episode was unavailable in 5 TTP cases.

**Table 5:** Age at onset according to the rs6903608 genotype.

rs6903608 Genotype	Age at Onset (Mean, Range)	Mean Difference, 95% CI (Years)
TT (*n* = 20)	50 (20 to 76)	Reference
CT (*n* = 67)	45 (17 to 79)	4, −3 to 12
CC (*n* = 74)	42 (11 to 73)	7, 0 to 14

rs6903608 genotype: TT, homozygous reference; CT, heterozygous; CC homozygous risk. Mean difference and 95% CI were calculated using an independent samples *t*-test. CI, confidence interval; IQR, interquartile range.

**Table 6:** Follow-up characteristics of iTTP patients included in the cohort study.

	All iTTP Patients (*n* = 153)	TT (*n* = 18)	CT (*n* = 66)	CC (*n* = 69)
Median follow-up time, years (95% CI) *	4.9 (3.7 to 6.1)	3.6 (0.0 to 7.5)	5.8 (5.0 to 6.7)	4.0 (2.9 to 5.1)
Relapse n (%)	44 (29)	3 (17)	13 (20)	28 (41)
Death n (%)	3 (2)	2 (12)	0	1 (1)
Cancer n (%)	3 (2)	0	2 (3)	1 (1)
Censored alive at the end of study, n (%)	92 (60)	12 (67)	48 (73)	32 (46)
Censored alive lost to follow-up, n (%)	11 (7)	2 (11)	3 (5)	6 (9)

CI, confidence interval; iTTP, immune-mediated thrombotic thrombocytopenic purpura. * Estimated by the inverse Kaplan–Meier method.

**Table 7:** Cumulative incidence of relapse at 2, 4 and 6 years of follow up and at the end of the study.

	Cumulative Incidence of Relapse, % (95% CI)			
rs6903608 Genotype	at 2 Years	at 4 Years	at 6 Years	at End of Study
TT (*n* = 18)	13 (8 to 22)	13 (8 to 22)	30 (23 to 39)	30 (23 to 39)
CT (*n* = 66)	12 (8 to 17)	22 (16 to 29)	22 (16 to 29)	33 (23 to 46)
CC (*n* = 69)	23 (16 to 32)	46 (36 to 57)	46 (36 to 57)	76 (44 to 97)

## Supplementary Materials

The following is available online at https://www.mdpi.com/2077-0383/9/10/3379/s1, Supplemental Table S1: Concomitant autoimmune diseases and infectious triggers reported in the enrolled iTTP patients; Supplemental Table S2: Impact of rituximab on the association between variant rs6903608 and iTTP relapse; Figure S1: Schematic representation of the possible scenarios of association between HLA variant rs6903608 and iTTP.
